# Dietary polyphenol assessment for aging research

**DOI:** 10.18632/aging.206252

**Published:** 2025-05-13

**Authors:** Ryan Bradley, Kara Fitzgerald, Romilly Hodges, Jamie L. Villanueva

**Affiliations:** 1 Herbert Wertheim School of Public Health and Human Longevity Sciences, University of California, San Diego, CA 92093, USA; 2 Institute for Functional Medicine, WA 98003, USA; 3 College of Nutrition, Sonoran University, Tempe, AZ 85282, USA; 4 School of Nursing, University of Washington, Seattle, WA 98195, USA; 5 Helfgott Research Institute, National University of Natural Medicine, Portland, OR 97201, USA

**Keywords:** polyphenols, phytonutrients, DNA methylation, epigenetics, aging

There is a growing need to characterize and quantify dietary polyphenol intake across populations and samples using standardized and validated methods, as well as to understand intake in the context of preventive or therapeutic amounts.

In our recently published paper examining the effect of variables within a DNA-methylation targeted multi-modal intervention from our pilot study, a grouping of foods characterized as polyphenolic modulators of DNA methylation had the most substantial epigenetic age attenuation effects of various dietary and lifestyle factors [1]. This finding is supported by Yaskolka Meir and colleagues in their analysis of the DIRECT PLUS trial and others including Quach and colleagues in their analysis of lifestyle factors that influence epigenetic clocks [2, 3]. Dietary polyphenols exert additional, pleiotropic effects via the hallmarks of aging such as mitochondrial function, inflammation, DNA repair, autophagy, and cellular damage prevention [4, 5]. Many appear to be dependent on microbial activation, highlighting a likely relevant dependency on gut microbiota composition [5].

However, the characterization and quantification of polyphenol intake in epidemiology and trial design have been heterogeneous, making comparison across studies challenging. Estimations of polyphenol intake have used the Phenol Explorer database (http://phenol-explorer.eu/) [6–8], liquid chromatography studies of specific foods [3], urinary polyphenol excretion [3, 9], proxy biomarkers such as serum or skin carotenoids [2, 10] and serum folate [11, 12], and food frequency questionnaires that capture polyphenol-dense foods [11].

Target preventive or therapeutic intake ranges have also not been well characterized. Population studies in Italy, France, Poland, and Greece have estimated mean background polyphenol intake to range from 664 to 1905 mg/d [6–8, 13], but this wide range may in part reflect different calculation methods. Living in institutional settings has been associated with reduced polyphenol intake [14], which has relevance for improving older adult care. Dose-dependent benefits of polyphenols were observed in the PREDIMED cohort where the highest quintile consumed a mean of 1235 mg/d [15]. In the DIRECT PLUS trial, the additional 1240 mg/d polyphenols on top of a baseline diet were associated with greater epigenetic age attenuation in the Li and Hannum epigenetic age clocks [3]. In our original pilot study, an estimated 2908 mg/d polyphenols from the DNA-methylation targeted dietary protocol (unpublished data using the Phenol Explorer database) was the factor most associated with reductions in the specimen-appropriate (saliva was used) Horvath 2013 multi-tissue epigenetic age clock [1, 11].

Among the scientific community, including those of us engaged in studying healthy aging, developing standardized and validated methods for assessing and characterizing polyphenol intake and contextualizing intake ranges is needed to support future research.
